# Supplementary material for: Allelopathic Potential of Artemisia absinthium and Artemisia vulgaris from Serbia: Chemical Composition and Bioactivity on Weeds
Source: Plants (Basel). 2025 May 30;14(11):1663. doi: 10.3390/plants14111663 (PMC12158117; doi:10.3390/plants14111663)
Supplement: Supplementary file 1 [file plants-14-01663-s001.zip › plants-3634849-supplementary.pdf]

Table S1. The complete chemical composition of *A. absinthium* and *A. vulgaris* essential oil

No.	Component	[a]RI_{EXP}	[b]RI_{LIT}	Content (%)	
				Artemisia absinthium	*Artemisia vulgaris*
1	(3Z)-hexenol	796	797	n.d.*	0.09±0.00
2	(2E)-hexenol	853	854	0.13±0.01	0.86±0.03
3	Tricyclene	920	921	n.d.	0.11±0.01
4	α-thujone	925	924	n.d.	0.53±0.02
5	Sabinene	969	969	3.94±0.09	1.14±0.05
6	β-pinene	972	974	0.21±0.01	0.12±0.01
7	Myrcene	988	988	1.77±0.05	2.79±0.07
8	α-phellandrene	1001	1002	0.98±0.04	n.d.
9	α-terpinene	1013	1014	0.19±0.00	0.32±0.02
10	p-cymene	1020	1020	2.19±0.06	0.85±0.04
11	o-cymene	1023	1022	0.35±0.01	0.13±0.00
12	1,8-cineole	1026	1026	0.40±0.02	0.82±0.03
13	(Z)-β-ocimene	1031	1032	0.57±0.03	n.d.
14	(E)-β-ocimene	1044	1044	0.11±0.00	0.25±0.01
15	γ-terpinene	1053	1054	0.52±0.02	0.94±0.04
16	cis-linalool oxide	1068	1067	0.12±0.00	n.d.
17	artemisinic alcohol	1082	1080	n.d.	0.27±0.01
18	terpinolene	1085	1086	0.17±0.01	n.d.
19	6,7-epoxymyrcene	1092	1090	n.d.	0.19±0.01
20	linalool	1094	1095	4.17±0.17	n.d.
21	trans-sabinene hydrate	1097	1098	0.20±0.01	0.38±0.01
22	nonanal	1101	1100	0.57±0.02	n.d.
23	cis-rose oxide	1105	1106	0.09±0.00	n.d.
24	1-octen-3-yl acetate	1109	1110	0.43±0.02	n.d.
25	6-camphenol	1111	1111	n.d.	0.07±0.00
26	trans-thujone	1112	1112	18.90±0.86	n.d.
27	cis-p-menth-2-en-1-ol	1117	1118	0.09±0.00	n.d.
28	cis-ocimene epoxide	1127	1128	7.88±0.42	n.d.
29	cis-p-mentha-2,8-dien-1-ol	1132	1133	0.16±0.00	n.d.
30	iso-3-thujanol	1134	1134	0.46±0.02	0.08±0.00

31	trans-verbenol	1140	1140	n.d.	0.09±0.00
32	sabina ketone	1153	1154	0.14±0.01	n.d.
33	isoborneol	1156	1155	n.d.	0.29±0.01
34	pinocarvone	1160	1160	0.66±0.02	n.d.
35	cis-chrysanthenol	1161	1160	n.d.	0.26±0.01
36	4-terpineol	1173	1174	1.03±0.09	0.58±0.03
37	p-cymen-8-ol	1178	1179	0.75±0.04	n.d.
38	α-terpineol	1185	1186	0.12±0.01	0.08±0.00
39	methyl-salicylate	1189	1190	0.11±0.01	n.d.
40	myrtenol	1195	1194	0.13±0.00	0.11±0.00
41	cis-piperitol	1196	1195	0.17±0.01	n.d.
42	fragranol	1214	1214	0.13±0.01	n.d.
43	bornyl formate	1221	1222	0.25±0.02	n.d.
44	nerol	1226	1227	0.17±0.01	n.d.
45	(3Z)-hexenyl-2-methylbutanoate	1228	1229	0.24±0.02	n.d.
46	pulegone	1234	1233	0.20±0.01	n.d.
47	trans-chrysanthenyl acetate	1234	1235	n.d.	0.13±0.01
48	cuminaldehyde	1238	1238	1.52±0.06	0.08±0.00
49	(Z)-anethole	1248	1249	0.20±0.01	n.d.
50	geraniol	1248	1249	n.d.	0.15±0.01
51	linalyl acetate	1255	1254	0.10±0.00	n.d.
52	cis-chrysanthenyl acetate	1262	1261	0.11±0.01	7.17±0.16
53	geranial	1263	1264	n.d.	0.08±0.00
54	iso-3-thujanol acetate	1268	1267	n.d.	0.09±0.01
55	perillaldehyde	1269	1269	0.12±0.01	n.d.
56	(3Z)-hexenyl valerate	1279	1279	n.d.	0.28±0.01
57	bornyl acetate	1285	1284	1.03±0.08	0.77±0.03
58	lavandulyl acetate	1289	1288	0.29±0.02	n.d.
59	trans-sabinyl acetate	1290	1289	0.10±0.01	0.09±0.00
60	carvacrol	1298	1298	n.d.	0.18±0.01
61	undecanal	1304	1305	n.d.	0.08±0.00
62	cis-2,3-pinanediol	1317	1318	0.09±0.01	n.d.
63	myrtenyl acetate	1325	1324	n.d.	0.09±0.01
64	presilphiperfol-7-ene	1333	1334	n.d.	0.15±0.01
65	trans-carvyl acetate	1339	1339	n.d.	0.09±0.00

66	α-cubebene	1346	1345	n.d.	1.08±0.05
67	7-epi-silphiperfol-5-ene	1345	1345	0.10±0.00	n.d.
68	citronellyl acetate	1350	1350	0.15±0.01	n.d.
69	eugenol	1355	1356	0.64±0.02	0.11±0.01
70	cis-carvyl acetate	1366	1365	n.d.	0.09±0.01
71	cyclosativene	1370	1369	n.d.	1.48±0.09
72	α-ylangene	1372	1373	0.45±0.02	n.d.
73	α-copaen	1375	1374	0.10±0.01	0.17±0.01
74	geranyl acetate	1380	1379	0.49±0.03	n.d.
75	modheph-2-en	1381	1382	n.d.	1.09±0.06
76	trans-myrtanol acetate	1386	1385	0.45±0.02	n.d.
77	α-isocomene	1386	1387	n.d.	5.15±0.22
78	(Z)-jasmone	1392	1392	0.11±0.01	n.d.
79	ciperene	1397	1398	n.d.	2.54±0.09
80	sesquithujene	1405	1405	n.d.	0.07±0.01
81	β-isocomene	1408	1407	n.d.	0.86±0.03
82	β-caryophyllene	1416	1417	6.00±0.47	5.81±0.17
83	methyl-undecanoate	1424	1424	n.d.	0.29±0.01
84	cis-thujopsene	1429	1429	n.d.	0.77±0.03
85	trans-α-bergamotene	1432	1432	n.d.	0.31±0.01
86	aromadendrene	1439	1439	n.d.	0.15±0.01
87	(Z)-β-farnesen	1439	1440	0.12±0.01	n.d.
88	epi-β-santalen	1444	1445	0.40±0.02	0.29±0.01
89	α-humulen	1451	1452	0.41±0.03	3.43±0.08
100	(E)-β-farnesene	1455	1454	0.23±0.01	n.d.
101	β-santalene	1457	1457	n.d.	0.27±0.02
102	9-epi-(E)-caryophyllene	1465	1464	0.09±0.01	0.16±0.01
103	dehydro-sesquicineole	1470	1469	0.80±0.03	n.d.
104	4,5-di-epi-aristolochene	1471	1471	n.d.	1.42±0.06
105	γ-gurjunene	1476	1475	1.21±0.05	10.41±0.31
106	γ-humulene	1480	1481	n.d.	6.67±0.19
107	germacrene D	1483	1484	4.71±0.21	4.88±0.11
108	β-selinene	1491	1491	n.d.	4.86±0.07
109	(Z,E)-α-farnesene	1491	1491	0.24±0.01	n.d.

110	bicyclogermacrene	1500	1500	7.04±0.47	1.15±0.03
111	germacrene A	1509	1508	n.d.	0.83±0.04
112	lavandulyl isovalerate	1509	1509	0.74±0.03	n.d.
113	geranyl isobutyrate	1514	1514	0.14±0.01	n.d.
114	α-dehydro-ar-himachalene	1515	1514	0.13±0.01	n.d.
115	δ-kadinen	1522	1522	n.d.	1.94±0.05
116	davana etar izomer 3	1535	1535	n.d.	0.15±0.01
117	α-cadinene	1537	1537	0.11±0.00	n.d.
118	cis-sesquisabinene hydrate	1541	1542	n.d.	0.19±0.01
119	cis-cadinene ether	1553	1552	n.d.	0.92±0.03
120	(E)-nerolidol	1560	1561	n.d.	0.18±0.01
121	davanone B	1563	1564	0.31±0.02	n.d.
122	palustrol	1567	1567	0.48±0.03	n.d.
123	germacrene D-4-ol	1573	1574	5.35±0.39	n.d.
124	caryophyllene oxide	1581	1582	n.d.	3.75±0.14
125	neryl 2-methyl-butanoate	1584	1584	3.23±0.11	n.d.
126	davanone	1587	1587	1.04±0.07	5.62±0.20
127	fokienol	1597	1596	n.d.	0.42±0.02
128	geranyl 2-methyl-butyrate	1601	1601	0.97±0.04	n.d.
129	α-humulene epoxide II	1608	1606	n.d.	0.27±0.01
130	geranyl isovalerate	1607	1606	0.50±0.02	n.d.
131	β-himachalene oxide	1615	1615	n.d.	1.74±0.06
132	Junenol	1617	1618	0.35±0.01	n.d.
133	α-acorenol	1632	1632	0.30±0.02	0.18±0.01
134	epi-α-muurolol	1641	1640	n.d.	0.25±0.01
135	α-muurolol	1645	1644	n.d.	0.14±0.00
136	β-eudesmol	1650	1649	0.28±0.01	0.27±0.02
137	α-cadinol	1653	1652	n.d.	0.17±0.01
138	β-bisabolol oxide B	1655	1656	0.26±0.02	n.d.
139	selin-11-en-4-α-ol	1659	1658	n.d.	0.89±0.06
140	cis-calamenen-10-ol	1661	1660	n.d.	0.47±0.02
141	intermedeol	1664	1665	0.29±0.01	n.d.
142	β-bisabolol	1675	1674	n.d.	0.88±0.05
143	α-bisabolol	1685	1685	n.d.	0.16±0.01

144	(Z)-α-trans-bergamotol	1690	1690	n.d.	0.75±0.03
145	pentadecanal	1714	1715	n.d.	0.66±0.03
146	(2Z,6E)-farnesol	1722	1722	n.d.	0.19±0.01
147	guaiol acetate	1726	1725	n.d.	0.25±0.01
148	(2E,6E)-farnesal	1738	1740	0.47±0.02	n.d.
149	γ-costol	1744	1745	n.d.	0.26±0.02
150	7,14-anhydroamorph-4,9-dien	1756	1755	n.d.	0.21±0.01
151	α-costol	1772	1773	0.22±0.01	0.15±0.01
152	Hexadecanal	1831	1830	n.d.	0.13±0.01
153	hexahydro-farnesyl aceton	1843	1843	0.18±0.01	n.d.
154	(2E,6E)-farnesyl acetate	1843	1845	n.d.	0.29±0.02
155	(Z,Z)-farnesyl acetone	1858	1860	n.d.	0.14±0.01
156	(Z)-nuciferyl propanoate	1894	1893**	0.14±0.01	n.d.
157	(5E,9E)-farnesyl acetone	1912	1913	0.19±0.01	n.d.
158	geranyl benzoate	1957	1958	n.d.	1.85±0.04
159	geranyl-α-terpinene	1961	1962**	1.51±0.06	n.d.
160	hexadecyl acetate	2004	2003	2.13±0.11	n.d.
161	13-epi-manoyl oxide	2010	2009	3.27±0.13	n.d.
162	isobergapten	2034	2033	0.14±0.01	n.d.
163	methyl-linoleate	2095	2095	0.29±0.02	0.22±0.01
164	methyl-octadecanoat	2125	2124	n.d.	0.22±0.02
165	Linoleic acid	2133	2132	n.d.	0.21±0.01
	Total identified (%)			99.02	99.24
	Monoterpene hydrocarbons			11.00	7.18
	Oxygenated monoterpenes			40.78	12.23
	Sesquiterpene hydrocarbons			21.34	57.07
	Oxygenated sesquiterpenes			16.24	17.76
	Diterpene hydrocarbons			1.51	-
	Oxygenated diterpenes			3.27	-
	Phenylpropanoids			0.84	0.11
	Phenols			0.11	-
	Other			3.93	4.89

[a]RI_{EXP}-Retention Indexes experimentally determined (calculated relative to C6-C28 n-alkanes on the DB-5 column); [b]RI_{LIT} - Retention Indexes - literature data (Adams, 2007); *n.d - not detected; ** Retention Indexes - NIST (National Institute of Standards and Technology, U. S. Department of Commerce) database.

Table S2. Effects of different concentrations of *A. absinthium* and *A. vulgaris* plant extracts on seed germination and seedling growth of *A. retroflexus* and *S. viridis*

Parameters	Control	Plant extract concentration					
		0.25%	0.50%	0.75%	1.00%	2.50%	5.00%
**Plant extract of *Artemisia absinthium***							
Amaranthus retroflexus							
Seed germination (%)	95.33±6.41 **a**	87.33±9.93 **b**	54.00±7.04 **c**	20.67±3.93 **d**	2.00±3.35 **e**	0.00±0.00 **e**	0.00±0.00 **e**
Shoot length (cm)	2.93±0.64 **b**	3.32±0.48 **a**	2.45±0.46 **c**	1.25±0.30 **d**	0.47±0.15 **e**	0.00±0.00 **e**	0.00±0.00 **e**
Radicle length (cm)	2.47±0.49 **a**	0.89±0.23 **b**	0.75±0.17 **c**	0.46±0.19 **d**	0.23±0.12 **e**	0.00±0.00 **e**	0.00±0.00 **e**
Seedling length (cm)	5.40±0.94 **a**	4.21±0.56 **b**	3.20±0.47 **c**	1.71±0.40 **d**	0.70±0.10 **e**	0.00±0.00 **e**	0.00±0.00 **e**
Setaria viridis							
Seed germination (%)	88.67±7.34 **a**	88.00±6.20 **a**	88.00±6.69 **a**	86.00±4.20 **a**	77.33±7.00 **b**	7.33±4.68 **c**	0.00±0.00 **d**
Shoot length (cm)	6.30±0.69 **a**	3.48±0.79 **b**	3.06±0.61 **c**	2.48±0.52 **d**	1.48±0.37 **e**	0.31±0.15 **f**	0.00±0.00 **f**
Radicle length (cm)	1.88±0.27 **a**	1.62±0.31 **b**	1.04±0.28 **c**	0.65±0.17 **d**	0.47±0.13 **e**	0.16±0.05 **f**	0.00±0.00 **g**
Seedling length (cm)	8.18±0.74 **a**	5.10±0.86 **b**	4.10±0.71 **c**	3.13±0.53 **d**	1.95±0.37 **e**	0.47±0.17 **f**	0.00±0.00 **g**
**Plant extract of *Artemisia vulgaris***							
Amaranthus retroflexus							
Seed germination (%)	95.33±6.41 **a**	90.67±7.00 **a**	63.33±3.01 **b**	35.33±4.68 **c**	18.67±3.27 **d**	0.00±0.00 **e**	0.00±0.00 **e**
Shoot length (cm)	2.93±0.64 **c**	3.95±0.64 **a**	3.48±0.52 **b**	2.51±0.44 **d**	1.46±0.36 **e**	0.00±0.00 **f**	0.00±0.00 **f**
Radicle length (cm)	2.47±0.49 **a**	1.03±0.27 **b**	0.62±0.18 **c**	0.50±0.13 **d**	0.40±0.12 **d**	0.00±0.00 **e**	0.00±0.00 **e**
Seedling length (cm)	5.40±0.94 **a**	4.98±0.68 **b**	4.11±0.57 **c**	3.00±0.46 **d**	1.86±0.43 **e**	0.00±0.00 **f**	0.00±0.00 **f**
Setaria viridis							
Seed germination (%)	88.67±7.34 **a**	92.67±5.89 **a**	92.00±5.06 **a**	92.00±2.53 **a**	69.33±4.13 **b**	23.33±5.89 **c**	0.00±0.00 **d**
Shoot length (cm)	6.30±0.69 **a**	3.18±0.48 **b**	2.68±0.43 **c**	1.76±0.32 **d**	1.34±0.25 **e**	0.29±0.08 **f**	0.00±0.00 **g**
Radicle length (cm)	1.88±0.27 **a**	0.91±0.19 **b**	0.51±0.13 **c**	0.36±0.08 **d**	0.29±0.06 **de**	0.22±0.06 **e**	0.00±0.00 **f**
Seedling length (cm)	8.18±0.74 **a**	4.09±0.56 **b**	3.19±0.47 **c**	2.12±0.34 **d**	1.62±0.25 **e**	0.51±0.12 **f**	0.00±0.00 **g**

Data are presented as mean±standard deviation. The differences were assessed using a one-way analysis of variance (ANOVA) with Fisher's least significant difference (LSD) test, $p<0.05$. The mean values marked with different letters (a, b, c, d, e, f, g) differ significantly from row to row ($p<0.05$).

Table S3. Effects of different concentrations of *A. absinthium* and *A. vulgaris* essential oil on seed germination and seedling growth of *A. retroflexus* and *S. viridis*

Parameters	Control	Plant extract concentration					
		0.01%	0.025%	0.05%	0.10%	0.25%	0.50%
**Essential oil of *Artemisia absinthium***							
Amaranthus retroflexus							
Seed germination (%)	95.33±6.41 **a**	94.67±4.84 **a**	88.00±8.00 **a**	80.00±7.16 **b**	78.67±6.53 **b**	49.33±6.02 **c**	12.67±6.41 **d**
Shoot length (cm)	2.86±0.50 **a**	2.80±0.63 **a**	2.43±0.44 **b**	1.62±0.33 **c**	0.74±0.20 **d**	0.26±0.13 **e**	0.11±0.02 **f**
Radicle length (cm)	2.28±0.39 **a**	2.15±0.45 **b**	1.64±0.35 **c**	1.22±0.22 **d**	0.73±0.17 **e**	0.32±0.13 **f**	0.11±0.03 **g**
Seedling length (cm)	5.14±0.73 **a**	4.95±0.84 **a**	4.07±0.63 **b**	2.84±0.49 **c**	1.47±0.29 **d**	0.58±0.24 **e**	0.22±0.04 **f**
Setaria viridis							
Seed germination (%)	94.00±5.51 **a**	92.00±4.38 **a**	88.00±5.66 **a**	88.00±10.12 **a**	77.33±6.53 **b**	37.33±6.53 **c**	0.00±0.00 **d**
Shoot length (cm)	6.08±0.92 **a**	4.69±0.88 **b**	3.12±0.65 **c**	1.30±0.35 **d**	0.35±0.13 **e**	0.11±0.04 **f**	0.00±0.00 **f**
Radicle length (cm)	1.88±0.38 **a**	1.82±0.36 **a**	1.65±0.30 **b**	0.77±0.19 **c**	0.33±0.11 **d**	0.11±0.03 **e**	0.00±0.00 **e**
Seedling length (cm)	7.96±1.09 **a**	6.51±1.10 **b**	4.77±0.81 **c**	2.06±0.44 **d**	0.67±0.20 **e**	0.22±0.04 **f**	0.00±0.00 **f**
**Essential oil of *Artemisia vulgaris***							
Amaranthus retroflexus							
Seed germination (%)	95.33±6.41 **a**	95.33±5.89 **a**	78.67±6.53 **b**	73.33±6.53 **b**	62.67±7.45 **c**	46.00±6.07 **d**	36.00±7.16 **e**
Shoot length (cm)	2.86±0.50 **a**	2.57±0.42 **b**	1.37±0.26 **c**	0.90±0.17 **d**	0.72±0.14 **e**	0.53±0.11 **f**	0.44±0.10 **f**
Radicle length (cm)	2.28±0.39 **a**	2.14±0.35 **b**	1.39±0.25 **c**	1.00±0.17 **d**	0.75±0.14 **e**	0.53±0.11 **f**	0.42±0.09 **g**
Seedling length (cm)	5.14±0.73 **a**	4.71±0.61 **b**	2.76±0.41 **c**	1.90±0.30 **d**	1.47±0.25 **e**	1.06±0.21 **f**	0.86±0.17 **g**
Setaria viridis							
Seed germination (%)	94.00±5.51 **a**	94.67±3.27 **a**	92.00±6.20 **a**	92.00±6.69 **a**	91.33±5.32 **a**	70.00±4.20 **b**	39.33±3.01 **c**
Shoot length (cm)	6.08±0.92 **a**	4.96±0.80 **b**	3.35±0.67 **c**	2.11±0.53 **d**	1.40±0.41 **e**	0.52±0.12 **f**	0.15±0.05 **g**
Radicle length (cm)	1.88±0.38 **b**	2.03±0.41 **a**	1.86±0.35 **b**	1.63±0.45 **c**	1.22±0.29 **d**	0.48±0.11 **e**	0.14±0.05 **f**
Seedling length (cm)	7.96±1.09 **a**	6.99±1.06 **b**	5.21±0.84 **c**	3.74±0.85 **d**	2.63±0.56 **e**	0.99±0.20 **f**	0.28±0.08 **g**

Data are presented as mean±standard deviation. The differences were assessed using a one-way analysis of variance (ANOVA) with Fisher's least significant difference (LSD) test, $p<0.05$. The mean values marked with different letters (a, b, c, d, e, f, g) differ significantly from row to row ($p<0.05$).

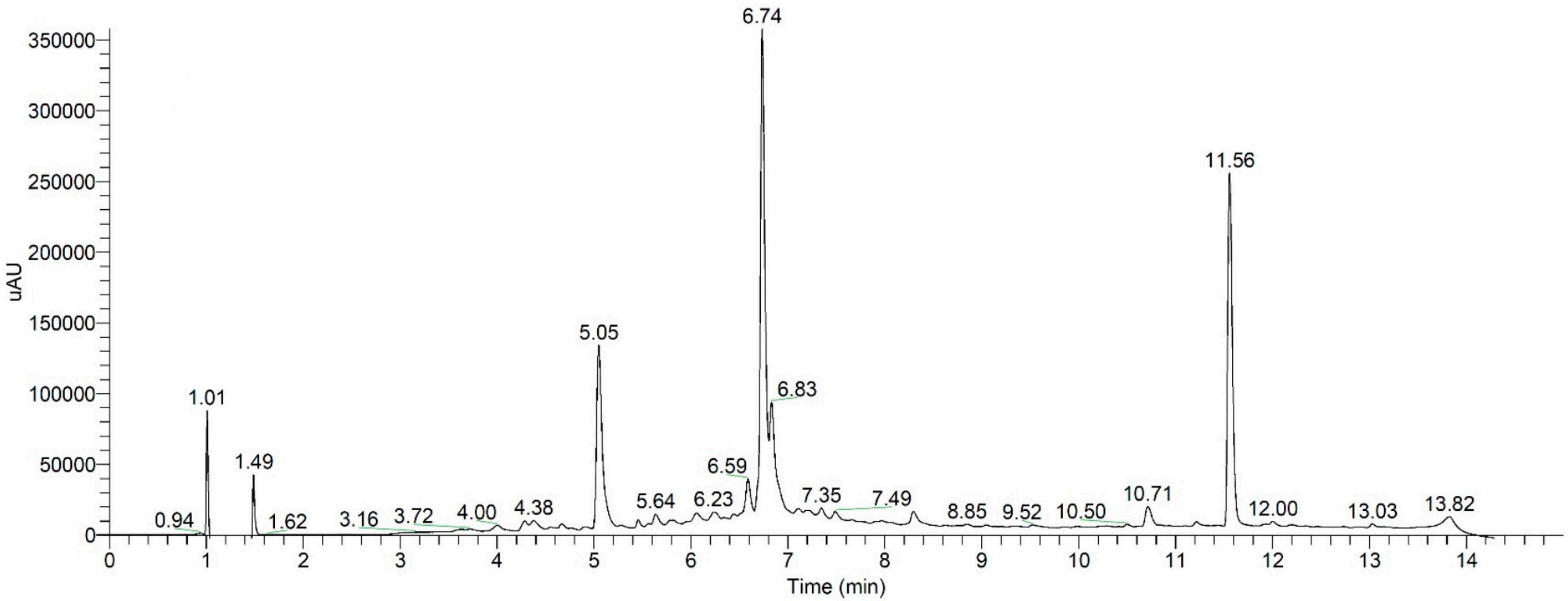

uAU
350000
300000
250000
200000
150000
100000
50000
0
6.74
11.56
5.05
1.01
6.83
1.49
6.59
0.94
1.62
3.16
3.72
4.00
4.38
5.64
6.23
7.35
7.49
8.85
9.52
10.50
10.71
12.00
13.03
13.82
0
1
2
3
4
5
6
7
8
9
10
11
12
13
14
Time (min)

(**a**)

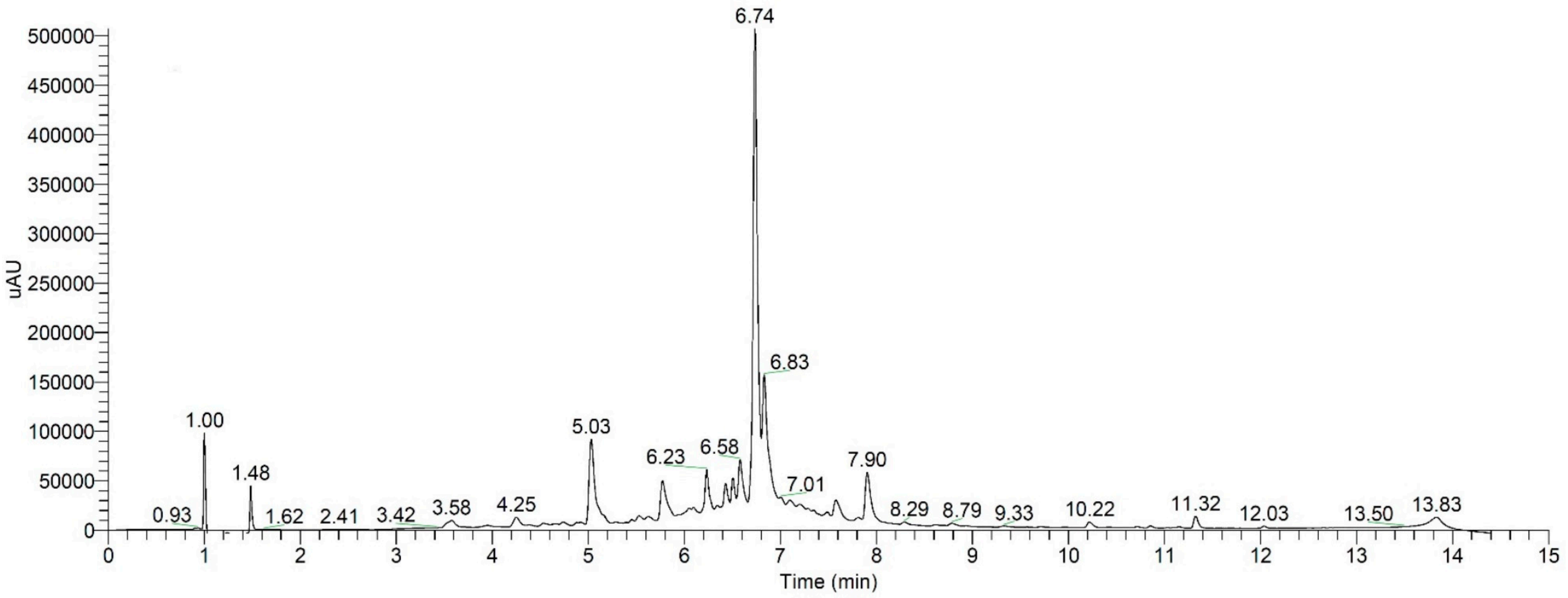

(**b**)

Figure S1. Chromatogram of *A. absinthium* (**a**) and *A. vulgaris* (**b**) plant extract

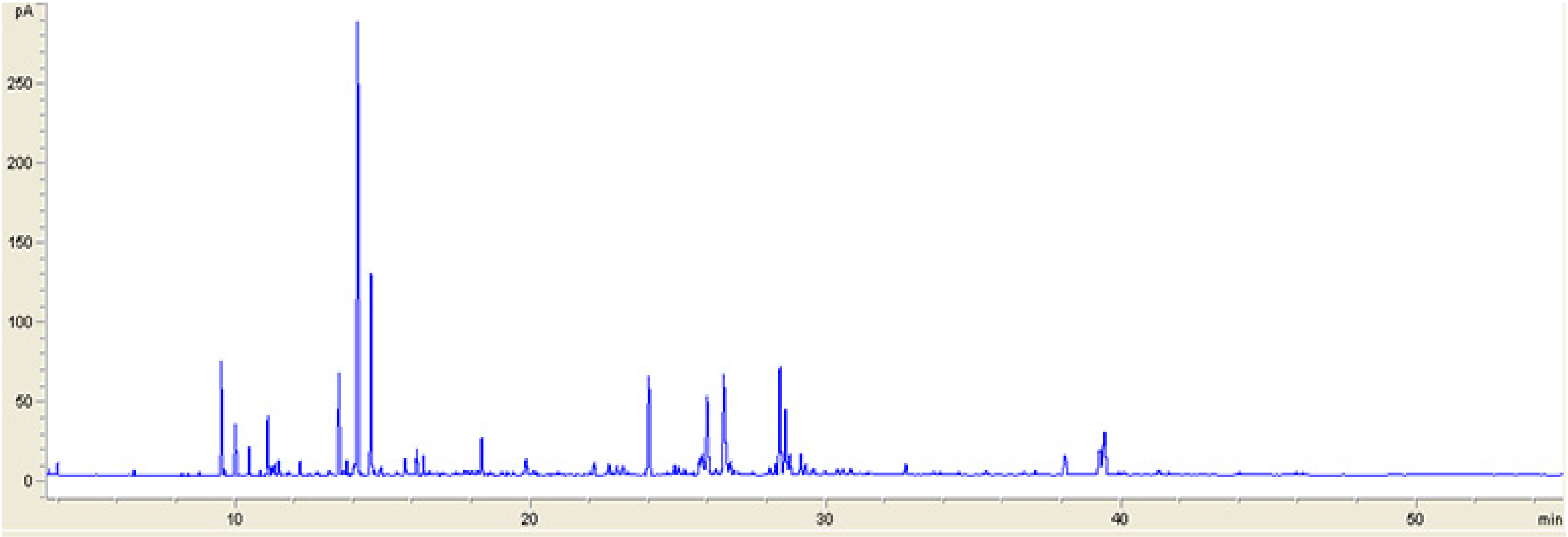

(**a**)

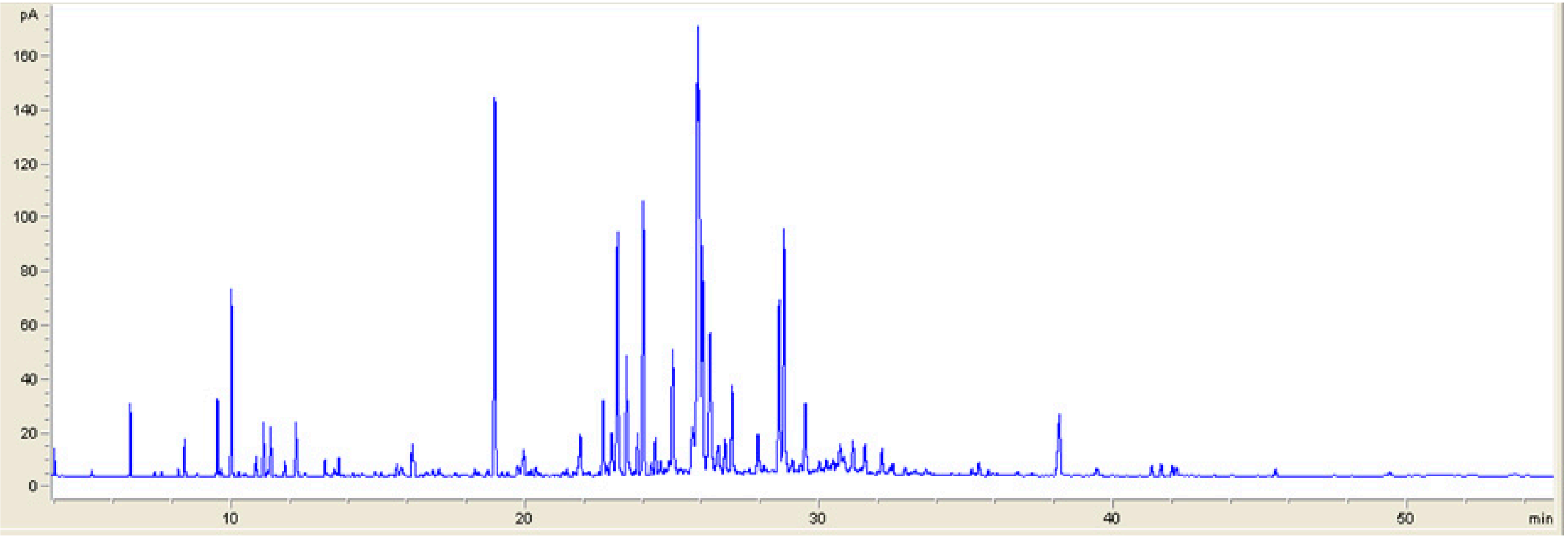

(**b**)

Figure S2. Chromatogram of *A. absinthium* (**a**) and *A. vulgaris* (**b**) essential oil
